# Coming to Terms: The Mechanisms of Overplus and Luxury Phosphorus Uptake for Polyphosphate Storage in Microalgae and Yeast

**DOI:** 10.1002/bit.70061

**Published:** 2025-09-06

**Authors:** Maxence Plouviez, Philipp Demling, Alexander Deitert, Jana Fees, Makarius Baier, Tobias Karmainski, Lars M. Blank, Benoit Guieysse

**Affiliations:** ^1^ Cawthron Institute Nelson New Zealand; ^2^ Institute of Applied Microbiology (iAMB), Aachen Biology and Biotechnology (ABBt), BioSC RWTH Aachen University Aachen Germany; ^3^ WSS Research Center “Catalaix” Aachen Germany; ^4^ BG Bioprocess Consulting Ltd Palmerston North New Zealand

**Keywords:** hyperaccumulation, luxury uptake, microalgae, overplus response, phosphate, phosphorus uptake, polyphosphates, yeast

## Abstract

Polyphosphate (polyP) synthesis is ubiquitous in organisms, including microorganisms such as microalgae and yeasts, playing a crucial role in phosphorus (P) metabolism (e.g., storage) and in other vital functions. Two mechanisms are broadly recognized for intracellular polyP accumulation: (i) The first involves a response to experiencing P repletion following a period of P depletion, often described as an overplus response because cells accumulate P at levels above what is considered physiologically “normal” if they had never experienced P depletion. PolyP accumulation likely occurs to maintain intracellular P homeostasis, with stored P supporting cell growth if extracellular P becomes depleted again. (ii) Contrarily, the second mechanism of polyP accumulation is not directly related to a change in P supply but is triggered by other conditions, such as the depletion of another nutrient. Because its purpose is less clear, this mechanism is often called “luxury P uptake.” Here, we briefly review concepts in polyP synthesis in microalgae and yeast as reasoning for differentiating the mechanisms described above based on environmental P availability, polyP chemistry, genetics, or cellular function. Based on this knowledge, we suggest a common terminology to be used across the different fields to allow comparability and avoid misunderstandings.

## Graphical abstract

Plouviez et al. give a perspective on the terminology used for describing the mechanisms of polyphosphate accumulation in microalgae and yeast. Their differentiation of mechanisms is based on environmental P availability, polyP chemistry, genetics, or cellular function as described in recent literature. Common practices in terminology usage and thorough analytical approaches are suggested to foster the comparability of polyphosphate research across different fields.

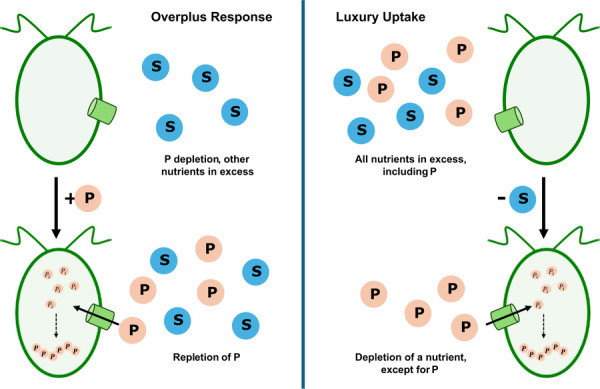

## 1 Introduction

Polyphosphates (polyP) are organic (e.g., inositol polyphosphates) or inorganic molecules (e.g., linear polyphosphate chains of 2–100s of phosphate (P_i_) groups linked by phosphoanhydride bonds) (Christ et al. 2020). PolyP are involved in multiple critical functions in microbial cells (Blank 2023; Kulaev 2005; Müller et al. 2022; Sanz‐Luque et al. 2020), and the ability of phosphorus‐accumulating microorganisms to store polyP has even led to the development of enhanced biological phosphorus removal (EBPR). This technology is broadly implemented to remove phosphorus (P) compounds during wastewater treatment (Nielsen et al. 2019; Oehmen et al. 2007; Roy et al. 2025; Slocombe et al. 2020). Further, polyP produced by microbes gained attention as a product of value, for eaxmple, for applications as food additives (Demling et al. 2024) or flame retardants (Chu et al. 2024). The molecular basis of polyP metabolism is relatively well understood in prokaryotes and yeasts, where polyP synthesis is driven by the enzymes polyP kinases (PPKs) and the vacuolar transport chaperone (VTC) complex, respectively (Austin and Mayer 2020; Azevedo and Saiardi 2017; Secco et al. 2012). Research on polyP metabolism has recently gained momentum in yeast and microalgae (Supporting information Figure S1) (Blank 2023; Plouviez and Brown 2024). For the latter, it has provided the scientific foundation to postulate that photoautotrophs could be used to upcycle P compounds from waste streams using sunlight as their primary energy source (Plouviez et al. 2023; Plouviez and Brown 2024). More research is, however, needed before microalgae‐based P management technologies can be engineered by reliably triggering polyP synthesis on demand in microalgae and cyanobacteria. Recent research suggests strong similarities in polyP synthesis between microalgae and yeast (e.g., VTC proteins, P_i_ sensing domains) (Cliff et al. 2023) and that P upcycling from waste streams can be transferred to a biotechnological process to synthesize polyP as a value‐added product of commercial interest (Christ and Blank 2019; Christ et al. 2020; Fees et al. 2023; Herrmann et al. 2023). Consequently, a knowledge transfer between research fields such as microalgae and yeast could lead to mutual benefits and foster the advancement of technologies for P upcycling into polyP. However, a thorough literature search indicated that different terminologies for the mechanisms involved in polyP synthesis are used for microalgae and yeasts (refer to Table 1 for definitions of key terminologies used in the field). Therefore, this article seeks to evidence the need to harmonize the terminology to optimize research focus in the field and identify areas of future research.

**Table 1:** Key terms and concepts used in P metabolism research.

Term/concept	Definition/rationale	Exemplary quotation
**Phosphate homeostasis**		
P‐specific response	The mechanisms of sensing and responding to change in P supply and requirement.	(Grossman and Aksoy (2015))
P acquisition (synonymous with accession, assimilation)	The general mechanism of P transport from the external environment into the cell and the subsequent use of this P.	(Persson et al. (2003)) (Secco et al. (2012))
Intracellular P reserves	P reserves can be found in different forms in different organelles. However, the bulk of P stores are in the form of polyphosphates.^a^	(Solovchenko et al. (2024))
P limitation	External P supply is restricted, but cells grow unaffected using cellular P reserves.	(Dyhrman (2016)) (Bossa et al. (2024))
P depletion	External P is lacking, and intracellular P reserves are low: growth is severely impacted.	(Plouviez et al. (2021))
P starvation	Both external and intracellular P reserves are lacking: Growth is impossible, and cells will ultimately die.	(Grossman and Aksoy (2015))
**Polyphosphate synthesis**		
P accumulation	Intracellular accumulation of P above a species‐specific threshold.	(Breus et al. (2012)) (Austin and Mayer (2020))
Overplus	P accumulation is “dependent” on external P supply and occurs when cells transition from being P‐starved to P‐repleted conditions.	(Plouviez et al. (2022))
Hyperaccumulation^b^	Synonym of overplus, i.e., often used for yeasts but rarely for algae.	(Deitert et al. (2024)) (Christ et al. (2020))
Over‐compensation or overshoot phenomenon	Synonyms of overplus	(Aitchison and Butt (1973)) (Cembella et al. (1984))
Surplus	Synonym of overplus	(Kulakovskaya et al. (2005))
Luxury P uptake/response	P accumulation is “independent” of external P and is triggered by a stress, generally the depletion of essential nutrients/factors such as sulfur or zinc.	(Slocombe et al. (2020))

^a^ While “normal” physiological conditions can be different between species, 1% intracellular P content has often been referred to as the norm (Plouviez et al. 2021). For example, P‐depleted *C. reinhardtii* has an intracellular P content of ~0.26 ± 0.12% as g_P_ g_CDW_^‐1^ (Plouviez et al. 2021). Intracellular P content as low as 0.03% has been reported (Whitton et al. 2016).

^b^ This term is often used for the accumulation of heavy metals in addition to the accumulation of P.

## 2 Key Concepts and Terminology

**Figure 1:**
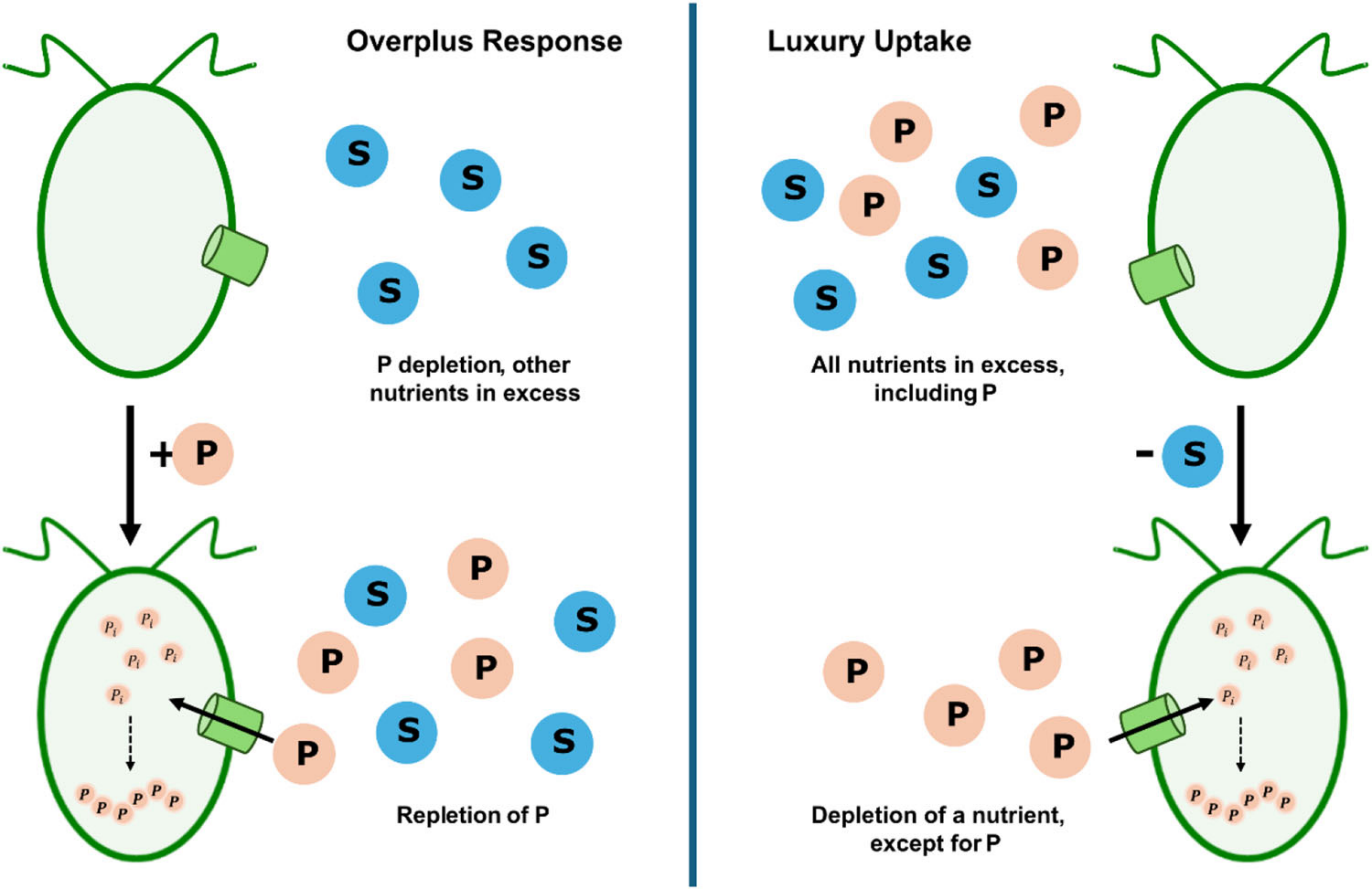
The mechanisms of overplus response and luxury uptake for intracellular polyphosphate accumulation. Overplus response: Intracellular accumulation of polyP in cells experiencing P repletion following a period of P depletion. Luxury uptake: PolyP synthesis in response to the lack of another key nutrient (e.g., sulfur) while P is available at all times. S: nutrient other than P, here for example sulfur.

The first attempt at differentiating the mechanisms responsible for polyP synthesis by the bacterium *Myxococcus xanthus* was made by Voelz et al. (1966) (Voelz et al. 1966), who stated the ‘Polymerization of inorganic phosphate can be induced by two distinct nutritional conditions: (1) by phosphate starvation with subsequent growth in an optimal phosphate concentration, a phenomenon designated as “Phosphat‐Ueberkompensation” or “polyphosphate overplus”; (2) by exhaustion of an essential nutrient’. These definitions have been extended to other polyP‐accumulating microorganisms and thus were generalized. Briefly, independent of the specific organism, the overplus response describes the intracellular accumulation of polyP in cells experiencing P repletion following a period of P depletion. In contrast, polyP synthesis in response to the lack of another key nutrient (e.g., sulfur) while P is available at all times is termed luxury uptake (Figure 1) (Björkman 2014; Dyhrman 2016; Slocombe et al. 2020; Solovchenko et al. 2019; Vasilieva et al. 2022; Zúñiga‐Burgos et al. 2024). Prominently, the terms have been used to describe c and, to a lesser extent, in yeasts, as the mechanism of luxury uptake is not distinctly defined, or the term is not used. Studies have been done on yeasts, reporting phosphate removal from nitrogen‐deficient cultures. However, P limitation was likely, with only 1 mM P_i_ in the pre‐cultivation medium, and an assessment of the degree of P limitation is missing (Breus et al. 2012; Breus et al. 2011). Independent of the field of research, a clear distinction of the mechanisms involved is often lacking, and the terms are sometimes used interchangeably, potentially leading to confusion about the exact mechanism observed. This highlights the need for an agreement on terminology between different taxonomic groups to improve knowledge transfer. Considering that the polyP biosynthetic pathways are related and similar phenomena are observed, this is highly relevant for microalgae and yeast (Cliff et al. 2023).

**Figure 2:**
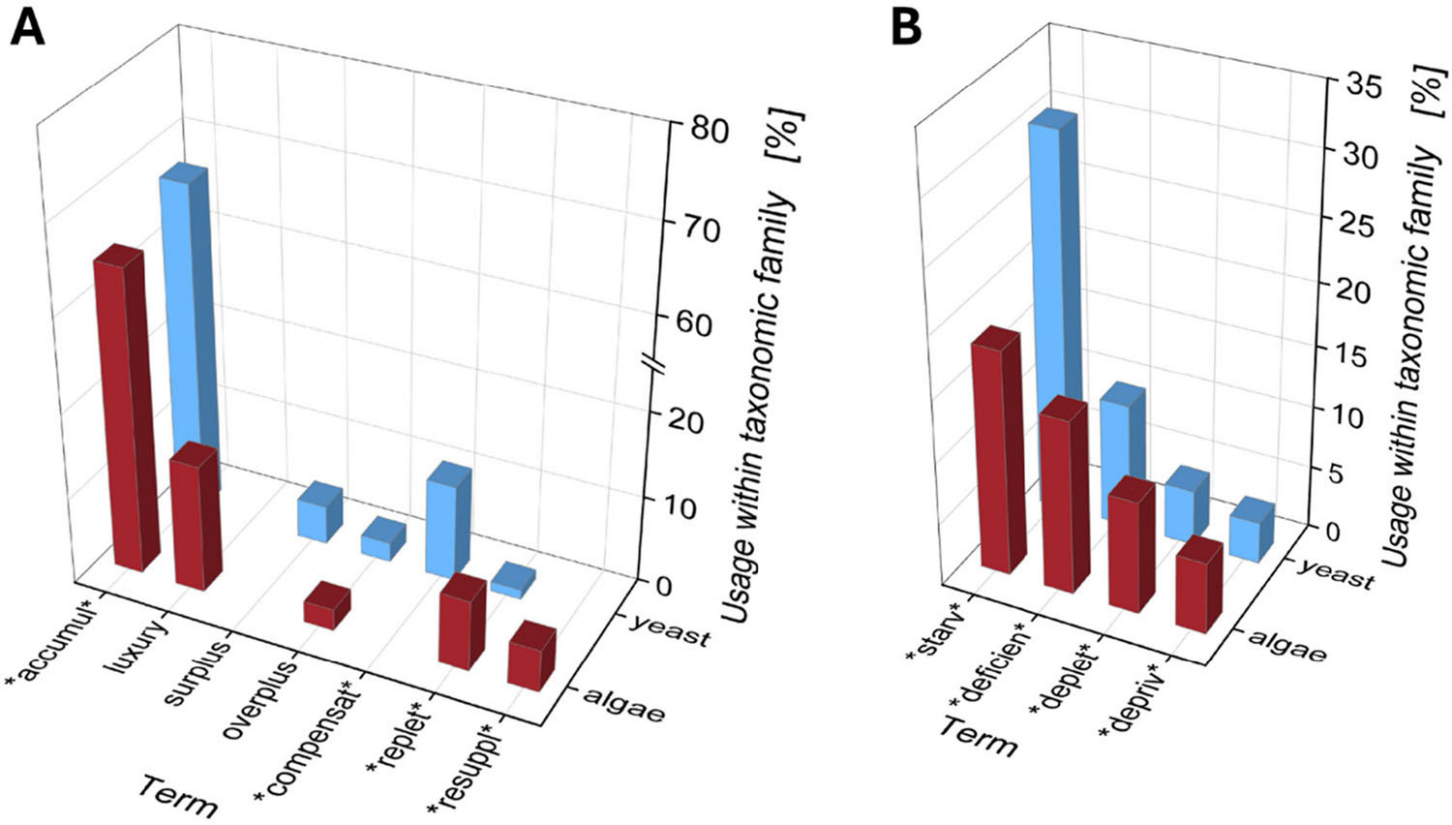
Usage of different terminology regarding polyP synthesis across different microalgae and yeasts. (A) Results for search terms related to P_i_ availability in excess. (B) Results for search terms related to the lack of P_i_. Data for yeast is represented in blue, data for algae is represented in red. The methodology applied for screening the polyP literature is described in the Supporting information.

An analysis of how frequently key terms related to polyP metabolism are used in yeast and microalgal research revealed time trends between fields (e.g., increase of polyP research in microalgae starting in the 2000s, Supporting information Figure S1) and a substantial increase in research outputs in the last 10 years (3.2‐fold more publications in 2001–2025 than in 1956–2000). However, this is true for many scientific fields, as the number of publications per year has drastically increased in recent years (Hanson et al. 2024). More importantly, there are notable differences between fields. While the terms “accumulation” and “starvation” are used for both yeast and microalgae, “compensation” and “surplus” are mainly used by the yeast community, whereas “repletion,” “re‐supplementation” and, especially, “luxury (P‐)uptake” are specific to microalgal research (Figure 2). It is unclear whether the terms are used based on cellular mechanisms or due to the “commonly” used yet ambiguous terminology in each respective field. To improve clarity and to provide a consistent terminology, the following sections discuss how terminology relates to characteristics of the “overplus” or “luxury P uptake” mechanisms and polyP synthesis, that is, extracellular P availability, the chemistry, content, and intracellular location of polyP, the genes and enzymes involved, or the cellular function of the polyP synthesized.

### 2.1 Extracellular P Availability

State‐of‐the‐art: P‐accumulating organisms are found in ecosystems where P concentration can differ 1000‐fold, from low ng/L levels in natural ‘P‐limited’ pristine lakes (e.g., Correll 1999) to mg/L in P‐laden wastewater effluents and their recipients (e.g., Longhurst et al. 2000). Microorganisms found in P‐limited environments must often cope with P supply frequently changing from P‐depleted to P‐repleted conditions (Björkman 2014) and, therefore, should theoretically accumulate P via an overplus response rather than luxury uptake. In contrast, microorganisms found in polluted streams or specific natural ecosystems, where P is constantly present at relatively high concentrations, should accumulate P via luxury uptake since there is no apparent advantage in expending energy to store a constantly available nutrient.

Notably, independently of the presence of specific organisms in the environments mentioned above, some of those organisms still have the ability to accumulate P as polyP in engineered environments with varying P supply. Baker's yeast is a prominent example of accumulating high amounts of polyP after undergoing P starvation and subsequent P repletion (Christ and Blank 2019; Christ et al. 2020), pointing towards an “overplus response.” Enhanced polyP synthesis in engineered environments can probably be initiated because the mechanism to cope with fluctuating P availability is conserved (Achbergerová and Nahálka 2011).

Implications for terminology: Identifying and naming polyP synthesis mechanisms based on P external availability appears to be the most relevant approach based on the historical definitions for the overplus response and luxury uptake mechanisms. However, while characterizing how cells experience P availability is generally straightforward in controlled laboratory experiments, this can be difficult to establish from observing complex natural ecosystems, especially within communities and micro‐environments experiencing rapid transient changes. In the latter case, there may be a need to look deeper inside cells to detect genetic regulations stimulated by extracellular P availability and the actual P starvation status of the cells, as discussed in the following sections.

### 2.2 Polyphosphate Chemistry, Structure, and Localization

State‐of‐the‐art: Both yeast and microalgae can accumulate up to 7%–8% P of their dry cell mass as polyP, primarily stored in acidocalcisome‐like vacuoles with high concentrations of mono‐ and divalent cations serving as counterions for the negatively charged polyP (Azevedo et al. 2015; Christ and Blank 2019; Diaz et al. 2009; Tsednee et al. 2019; Wang et al. 2023). In yeast, the polyP fraction has been well characterized to consist mainly of sodium, potassium, and magnesium as counterions (Christ et al. 2020; Fees et al. 2023; Herrmann et al. 2023). While magnesium has been proposed to be the predominant counterion in microalgae as well (Slocombe et al. 2023), in‐depth characterization is still needed. As for the P availability discussed in the previous paragraph, the counterion depends on the cultivation environment. The availability of cations in controlled bioprocesses designed explicitly for enhanced polyP production differs from natural or polluted environments. Regarding the localization of polyP, minor amounts are found in other organelles of eukaryotic microbes than the acidocalcisome‐like vacuoles, such as mitochondria, the endoplasmic reticulum, or the periplasmic space, and specifically for microalgae, in the chloroplast (Azevedo et al. 2020; Kulaev and Kulakovskaya 2000; Pestov et al. 2004; Sanz‐Luque et al. 2020). Independent of the mechanism involved (“overplus response“ or “luxury uptake”), comparable amounts of accumulated P (as P_i_) have been reported for studies focusing on microalgae (Plouviez and Brown 2024). Unfortunately, a thorough characterization that includes the chemistry (i.e., concentration and counterions) and the structure (i.e., chain length distribution and branching), combined with the localization of polyP, is lacking, as mentioned above. Therefore, identifying potential differences between overplus and luxury uptake based on biochemical characteristics remains uncertain.

**Figure 3:**
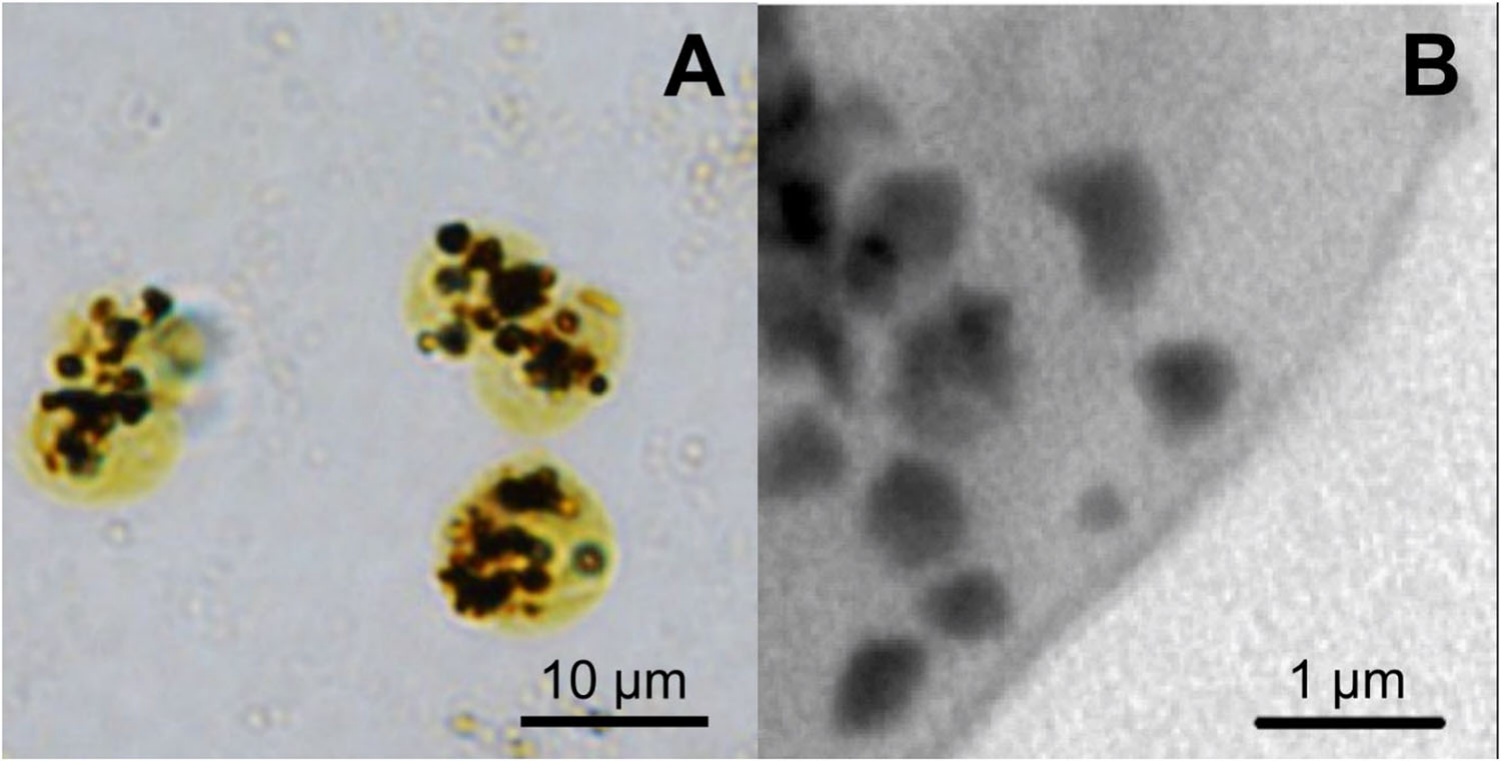
Lead‐sulfide staining specifically targets inorganic polyP and is used to visualize polyphosphate granules as dark deposits in cells. Exemplified in: (A) P repleted *Chlamydomonas reinhardtii* cells stained with lead‐sulfide and observed under an optical microscope (×100 oil immersion, scale bar = 10 µm). (B) STXM images of P repleted *Chlamydomonas reinhardtii* (scale bar = 1 µm), adapted with permission from Plouviez et al. 2024. Copyright 2024 American Chemical Society.

Implications for terminology: In our experience, microalgal polyP accumulation in granules becomes visible when P accumulates above 0.26 ± 0.12% as g_P_ g_CDW_^−1^ reported under P‐depleted conditions (Plouviez et al. 2023; Plouviez et al. 2021). Thus, distinguishing whether overplus response or luxury uptake is taking place could be to show that intracellular P accumulation is occurring above what is considered the ‘normal’ physiological level for a given organism. Beyond this standard threshold, the current literature does not support the view that the two mechanisms involved in polyP synthesis in microalgae can be distinguished based on their chemistry, structure, or cellular localization. In addition, even if structural differences existed, direct microscopic visualization can be imprecise (Figure 3), and identifying the intracellular repositories for polyP storage is practically challenging since purified fractions from the individual organelles are necessary, requiring advanced equipment or expertise (Goodenough et al. 2019). The development of precise analytical methods is therefore critically needed to link the different characteristics with the mechanisms and their respective functions.

### 2.3 Polyphosphate Metabolism, Genetics, and Regulation

**Figure 4:**
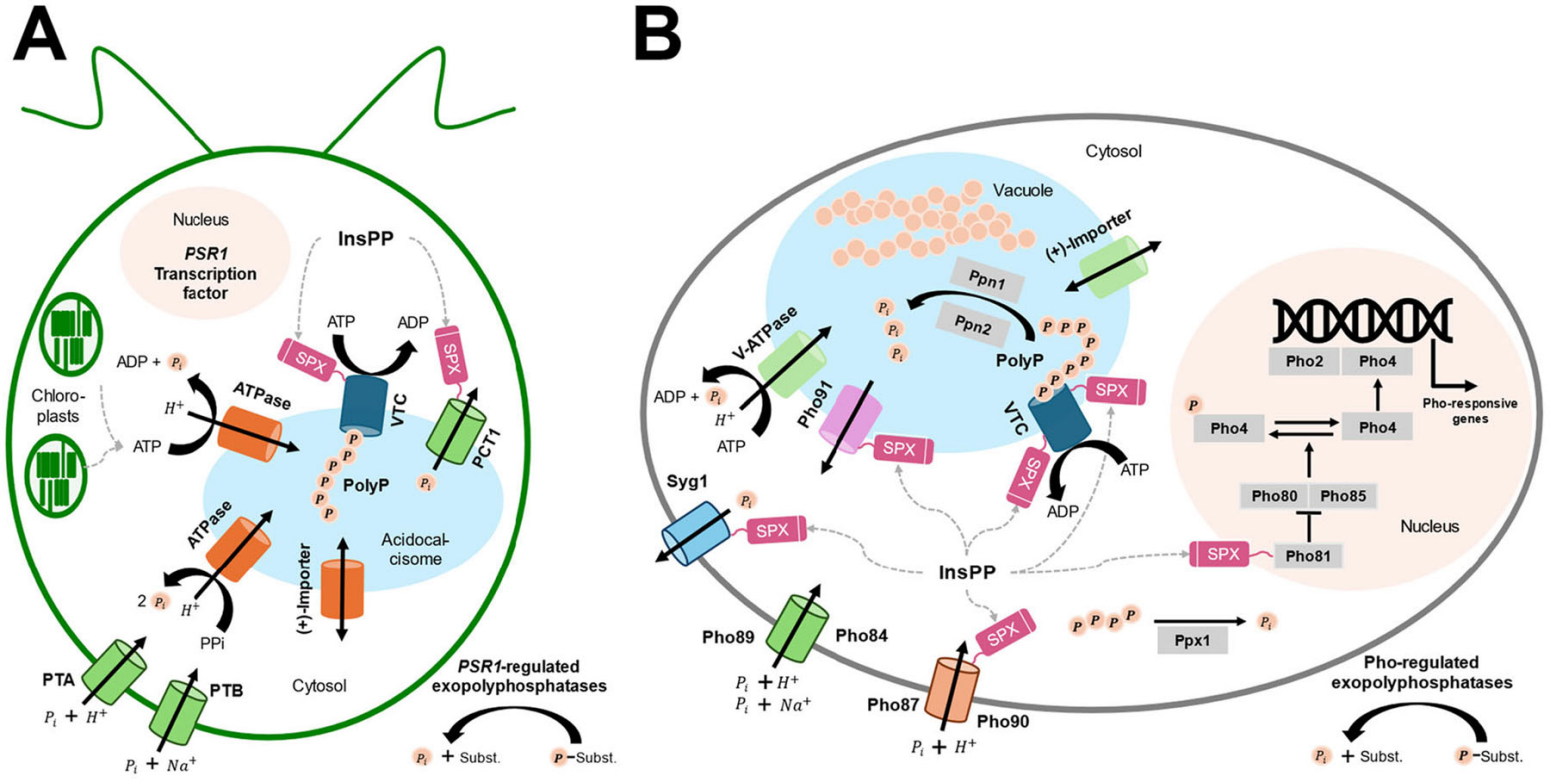
Metabolic pathways for P_i_ uptake and polyP synthesis in microalgae and yeast. Simplified for (A) microalgae (here for *C. reinhardtii*) and (B) yeast (here for *S. cerevisiae*). While the responses to changes in P_i_ availability involve several different genes, the overall responses between yeast and microalgae are similar. Controlled by the PSR1 transcription factor in microalgae or the PHO transcription pathway in yeast, genes encoding phosphatases and P_i_ transporters are upregulated during P_i_ depletion. This condition also upregulates the genes encoding for the proteins catalyzing polyP synthesis, the vacuolar transport chaperone complex (VTC). P_i_ is then used in key metabolic pathways and intracellular P_i_ homeostasis is regulated via binding of inositol pyrophosphates (InsPP) to the SPX regulatory domain of proteins (e.g., VTC). Refer to (Sanz‐Luque and Grossman 2023) and (Austin and Mayer 2020) for further details in microalgae and yeast, respectively. ADP, adenosine diphosphate; ATP, adenosine triphosphate; ATPase, adenosine triphosphatase; InsPP, inositol pyrophosphates; Pho81/80/85/4/2, PHO pathway, P_i_ signaling system regulating the gene expression in response to P_i_ availability; Pho84/89, high‐affinity P_i_ transporters; Pho87/90, low‐affinity P_i_ transporters; Pho91, vacuolar phosphate transporter; P_i_, inorganic phosphate; PP_i_, inorganic pyrophosphate; Ppn1, endopolyphosphatase 1; Ppn2, endopolyphosphatase 2; Ppx1, exopolyphosphatase 1; polyP, polyphosphate; *PSR1*, phosphate starvation response gene 1 (transcription factor); PTA, P_i_ transporter A; PTB, P_i_ transporter B; SPX, SYG1, PHO81, and XPR1 domain; Syg1, suppressor of yeast gpa1; V‐ATPase, vacuolar‐type ATPase; VTC, vacuolar transporter chaperone complex.

**Figure 5:**
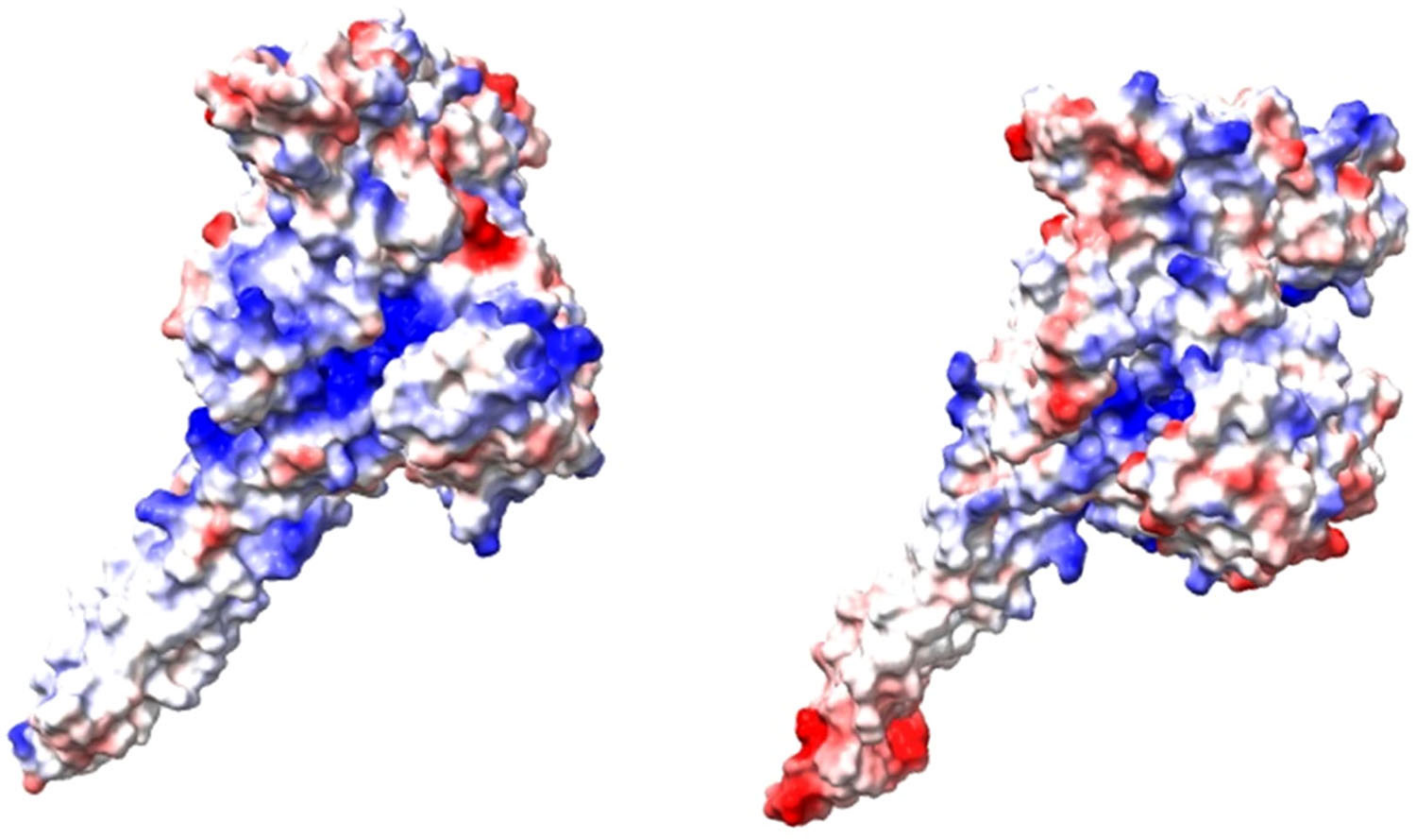
VTC4 models of *S. cerevisiae* (left) and *C. reinhardtii* (right). Generated using Alphafold3. The predicted models were further refined by structure equilibration and minimization in a TIP3 water box. Surface colors mapped to the electrostatic potential (−10: red to 10: blue KbT/e).

State‐of‐the‐art: The molecular pathways of P metabolism (Figure 4) have been well characterized in bacteria, yeast, and microalgae, where a decrease in P availability typically triggers the upregulation of genes required to recycle P, improving its acquisition via high‐affinity transporters (Hürlimann et al. 2009; Sanz‐Luque et al. 2020; Sanz‐Luque and Grossman 2023; Secco et al. 2012; Tiwari 2024). Reduced P availability also triggers yeast and microalgal cells to upregulate the genes encoding for their respective polyP synthesis proteins, forming the VTC complex (Plouviez et al. 2023; Plouviez et al. 2021; Secco et al. 2012). While the overall responses are similar, the genes involved differ even within eukaryotes. As previously reviewed, the multi‐level INPHORS (intracellular phosphate reception and signaling) pathway regulates the maintenance of phosphate homeostasis and has been most extensively studied in *S. cerevisiae* (Austin and Mayer 2020; Azevedo and Saiardi 2017). However, this pathway is not conserved throughout eukaryotic microbes, even in the relatively closely related yeast *Schizosaccharomyces pombe*, which lacks homologs of some key proteins and consequently exhibits a different transcriptomic response during P starvation compared to *S. cerevisiae* (Carter‐O'Connell et al. 2012). In comparison, the microalgal P metabolic pathway is controlled by the transcription factor phosphate starvation response 1 (Plouviez et al. 2021). For polyP synthesis, microalgae possess only homologs of the VTC1 and VTC4 subunits of the yeast *S. cerevisiae*. Hence, the VTC complexes, consisting of 3 x VTC1/1 x VTC2 or VTC3/1 x VTC4 in yeast, potentially differ in structure in microalgae. Nevertheless, the polyP polymerase activity of the microalgal VTC4 subunit has been demonstrated using repressed mutants as well as in silico (Figure 5), and the study of VTC4 models also evidenced the binding of inositol hexakisphosphate (InsP6) to the SPX domain of VTC4 from four microalgae species (Cliff et al. 2023). The presence of InsP6 in polyP granules of some microalgae (Plouviez et al. 2024) and further in silico evidence suggest that the activation of polyP synthesis in microalgae is similar to that of yeast (i.e. involving inositol pyrophosphates), particularly *S. cerevisiae*.

Implications for terminology: In yeast, polyP synthesis is performed by the VTC complex, consisting of different multimers according to the sub‐locations (i.e., endoplasmic reticulum or acidocalcisome) (Desfougères et al. 2016; Hothorn et al. 2009; Uttenweiler et al. 2007). While the exact pathway and related transcriptional responses differ between eukaryotes, regulating phosphate homeostasis is crucial for fundamental cell functions. To our knowledge, polyP synthesis always involves the VTC complex in microalgae, regardless of whether polyP synthesis occurs during an overplus response or luxury uptake. Therefore, the two mechanisms cannot be distinguished based on their polyP synthesis. Further, it is unknown if the regulation of microalgal polyP synthesis is similar during overplus response and luxury P uptake, as the different triggering mechanisms (P‐dependent vs. P‐independent) may suggest. Therefore, further research is needed in this area.

### 2.4 Cellular Function of Polyphosphate

State‐of‐the‐art: Various functions have been attributed to polyP in yeast and microalgae, from phosphorus storage to osmotic stress response, cell cycle progression, genomic stability, and posttranslational modifications of proteins (Azevedo et al. 2024; Azevedo et al. 2015; Azevedo and Saiardi 2016; Bru et al. 2016; Müller et al. 2019; Neville et al. 2024; Sanz‐Luque et al. 2020). PolyP synthesis and co‐localization into acidocalcisome‐like vacuoles has been linked with a cellular mechanism to overcome toxicity of high concentrations of extracellular and cytosolic P_i_ which has been demonstrated to inhibit cell growth (Gerasimaitė et al. 2014; Li et al. 2018). Here, short‐chain polyP might play a prominent role (Solovchenko et al. 2024), however further research is required. Although it has been suggested that polyP may be used as a source of energy, some authors have reported that polyP is not degraded when energy is limited in bacterial and microalgal cells. In addition, the energy stored in polyP is relatively small and can only fuel cell metabolism for a very short time (Harold 1966; Plouviez et al. 2022). This could explain why the energy penalty of polyP synthesis on growth appears to be insignificant, as suggested by an experiment conducted by Plouviez et al. (2023), in which light‐ (i.e., energy‐) limited microalgae grew at similar rates despite accumulating significantly different amounts of intracellular P (as % dry cell mass) under different P supply. A more likely function of polyP accumulation is to act as a P storage and a mechanism to maintain P homeostasis and reduce the adverse effects of high cytosolic phosphate (or polyP) concentrations, as suggested in yeasts (Desfougères et al. 2016; Gerasimaitė et al. 2014). A beneficial strategy for cells frequently experiencing P limitation would, therefore, be to expend a relatively minor amount of energy (< 1% of the energy contained in biomass [Plouviez et al. 2022]) to store a significant amount of P as “inert” polyP during periods of P abundance and later use these internal P stores to support growth during periods of P limitation or depletion. This type of evolutionary response is reminiscent of other “feast‐and‐famine” based strategies investigated for producing polyhydroxyalkanoates (PHAs) from food waste (Oliveira et al. 2017), removing P (Klein et al. 2022) or N (D. Li et al. 2021) from wastewater using aerobic granules, and reducing membrane fouling during wastewater treatment (Corsino et al. 2022).

As luxury P uptake is dependent on other nutrients and factors than P availability, polyP synthesis during luxury uptake should have a different function than P storage. However, it is currently unknown if luxury P uptake is the fortuitous consequence of an imperfect regulatory mechanism or if polyP is required by cells to enhance the management of the uptake and internal storage of other nutrients (e.g., utilizing negatively charged polyP to co‐store metallic cations). As such, the current terminology may be intrinsically misleading as it implies a “luxury” response that is not required by the cells.

Implications for terminology: The use of polyP as a P reserve may be logically associated with an overplus response, but this does not exclude polyP from other functions in P‐limited cells. In contrast, the function of polyP synthesis during luxury response is unclear and not necessarily exclusive of P storage. Additionally, the practical difficulty of inferring function solely from observing polyP synthesis and/or depletion in complex ecosystems (e.g., a dividing cell may split its polyP storage between itself and the daughter cell without using the stored polyP), the putative intracellular functions of polyP can currently not be used to distinguish between overplus response and luxury uptake.

## 3 Perspective

Based on the state of the art, it is currently impossible to identify the mechanism of polyP synthesis based on the genes and enzymes involved, nor is it possible to determine it based on the chemistry, quantity, and intracellular location of the polyP synthesized. While using polyP for P storage could indicate an overplus response, this function can be challenging to demonstrate and may not be specific to this mechanism. Therefore, focusing on the environmental conditions associated with polyP synthesis remains the best means to distinguish the mechanism involved. Further, this is easily transferable to other organisms, which exhibit the capability of accumulating polyP under similar conditions. We therefore propose that the term “P overplus response” should be used to specifically describe cells rapidly accumulating P following P repletion after experiencing a period of P depletion, whereas “luxury uptake” should only be used when a P depletion stage is not required before P accumulation and/or when P‐uptake is independent of the environmental P availability. In addition, both overplus response and luxury uptake should always be demonstrated by evidencing that P is intracellularly accumulated as polyP above the “normal” physiological level for a given organism. The superordinate term “accumulation” could be used regardless of the mechanisms or purpose of polyP production. Still, it should consistently be demonstrated by either an increase in intracellular P concentration over time or in relation to the external (environmental) P concentration. Notably, the P status of the cell (P depletion: P is limited but can be mobilized by using intracellular reserves; P starvation: no P or P reserves are available; Table 1) can substantially impact P assimilation and should therefore be differentiated as cultivation conditions or physiological states of the cell. The latter must be explicitly measured.

Further, we propose using standard metrics and precise descriptions of the measured parameters to enhance the comparability of the P amounts accumulated across different scientific fields and studies. At least, this should always include the percentage of P in the cell dry weight, thereby preventing incomparability introduced by different (analytical) representations of (poly)phosphates or P‐containing compounds (such as KPO_3_). Finally, further analytics should disclose if the P was accumulated as polyP, P_i_, or other P‐containing molecules. Reporting ratios are key to discovering potential similarities and differences in the mechanisms involved if more than one molecular compound was detected.

## Author Contributions

Maxence Plouviez drafted and edited the article and designed figures. Philipp Demling and Alexander Deitert contributed by drafting and editing the article, designing figures, and conducting literature research. Jana Fees and Lars M. Blank supported drafting and editing the article, while Makarius Baier and Tobias Karmainski assisted with drafting, editing, and literature research. Benoit Guieysse also contributed to drafting and editing the article.

## Conflicts of Interest

Lars M. Blank is a coinventor of the following patents: EP3886609A1 and EP4061768A1. Other than that, the authors declare no conflicts of interest.

## Supporting information

Plouviez Terms PolyP SuppMat1.

Plouviez Terms PolyP SuppMat2 revised.

## Data Availability

The data that supports the findings of this study are available in the supporting material of this article.
