# Supplementary material for: Coming to Terms: The Mechanisms of Overplus and Luxury Phosphorus Uptake for Polyphosphate Storage in Microalgae and Yeast
Source: Biotechnol Bioeng. 2025 Sep 6;122(12):3486–95. doi: 10.1002/bit.70061 (PMC12599490; doi:10.1002/bit.70061)
Supplement: Supplementary file 1 — Plouviez Terms PolyP SuppMat1. [file BIT-122-3486-s002.docx]

**Supplemental Material to:**

**Coming to terms: The mechanisms of overplus and luxury phosphorus uptake for polyphosphate storage in microalgae and yeast**

Maxence Plouviez^1^, Philipp Demling^2§^, Alexander Deitert^2^, Jana Fees^2^, Makarius Baier^2^, Tobias Karmainski^2^, Lars M. Blank^2,3^, Benoit Guieysse^4^

1 Cawthron Institute, Nelson, New Zealand

2 Institute of Applied Microbiology (iAMB), Aachen Biology and Biotechnology (ABBt), BioSC, RWTH Aachen University, Germany

3 WSS Research Centre ‘catalaix’

4 BG Bioprocess Consulting Ltd, New Zealand

§ Correspondence to:

Dr.-Ing. Philipp Demling, philipp.demling@rwth-aachen.de

**Methodology of the literature research**

Scopus (Elsevier) was used to perform a comprehensive literature search regarding the commonly used terms for polyphosphate biosynthesis in yeast and (micro-)algae research. The following search input was applied to scan the abstract of articles:

( *accumul* OR luxury OR surplus OR *overplus OR *compensat* OR *replet* OR *resuppl* OR *starv* OR *deplet* OR *deficien* OR *depriv* ) AND ( polyphosph* OR polyp OR poly-p ) AND ( yeast OR *alga* )

The search yielded 396 results (as of May 1^st^, 2025). The DOIs of each article, publication year, search terms appearing, the respective organism class, and the specific organism were transferred to an Excel sheet and enumerated. Articles unrelated to polyphosphate biosynthesis were marked as such and omitted subsequently. The resulting data was further processed, summing up totals per algae and yeast per year and in 5-year intervals. Further, the frequency of search terms used in total and used for yeast and algae, respectively, was calculated, as well as per year and 5-year intervals.


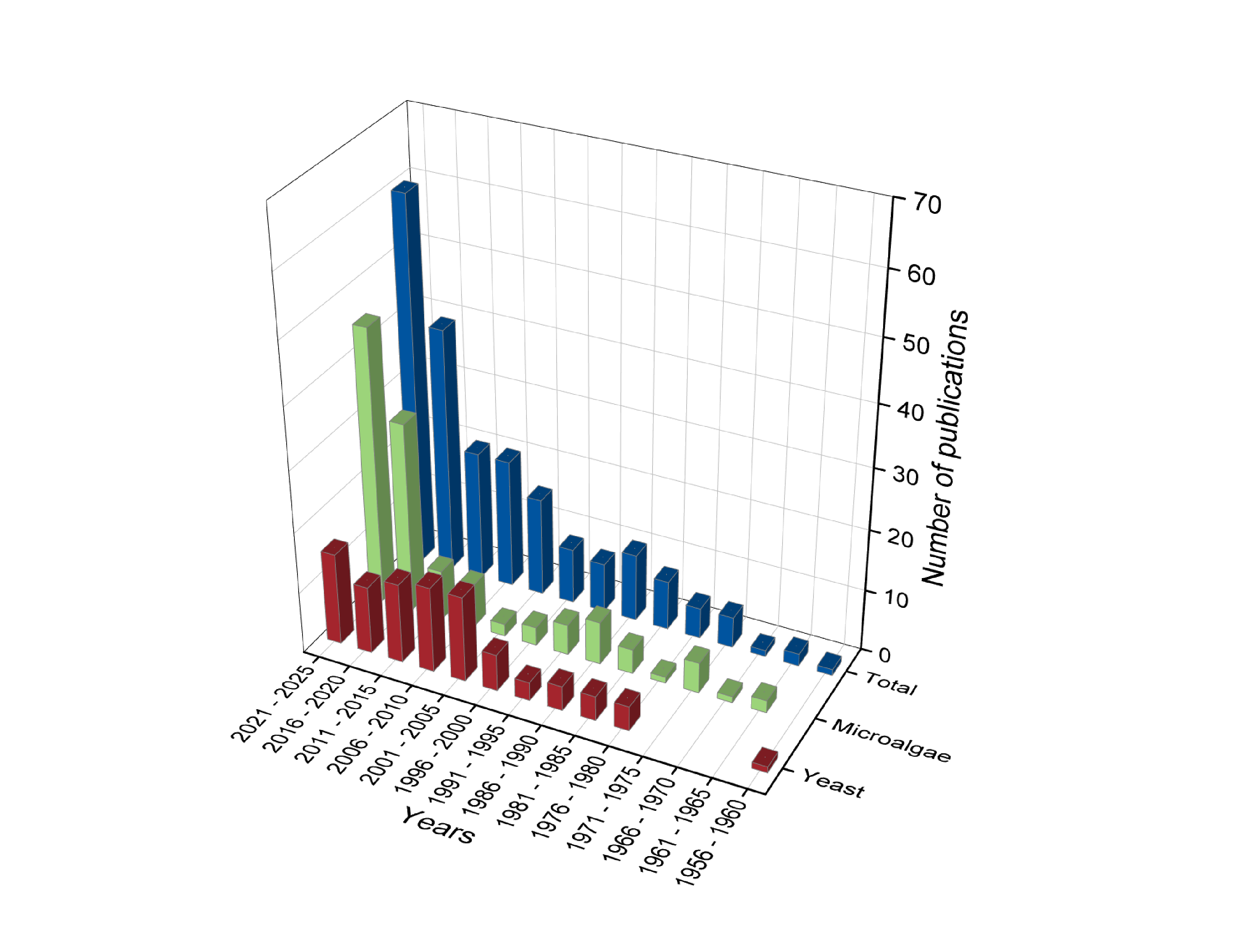


**Figure S1. Number of publications dealing with the biosynthesis of polyphosphate in yeast and microalgae from 1956 to 2025.** Individual years are consolidated in 5-year intervals.
